# Analysis of the effect of 6‐Hz binaural beats on electroencephalographic and autonomic parameters of healthy individuals: An exploratory study

**DOI:** 10.14814/phy2.70271

**Published:** 2025-05-22

**Authors:** Irene Scala, Jessica Marotta, Valerio Brunetti, Filippo Del Tedesco, Rikardo Xhemalaj, Pier Andrea Rizzo, Giovanni Frisullo, Eleonora Rollo, Maria Grazia Bocci, Edoardo Piervincenzi, Giacomo Della Marca, Riccardo Maviglia

**Affiliations:** ^1^ Università Cattolica del Sacro Cuore Rome Italy; ^2^ Dipartimento di Neuroscienze, Organi di Senso e Torace Fondazione Policlinico Universitario A. Gemelli IRCCS Rome Italy; ^3^ Dipartimento di Scienze dell'emergenza, Anestesiologiche e Della Rianimazione Fondazione Policlinico Universitario A. Gemelli IRCCS Rome Italy; ^4^ Istituto Nazionale per le Malattie Infettive L. Spallanzani INMI RICCS Rome Italy

**Keywords:** autonomic nervous system, binaural beats, eLORETA, lagged‐phase connectivity, theta frequency

## Abstract

Binaural beats (BB) are a non‐acoustic perception generated when two pure tones with a slight mismatch in frequency are presented separately to each ear. The aims of our study were to analyze the ability of 6‐Hz BB in entraining cortical activity, altering cortical connectivity, and influencing Autonomic Nervous System (ANS) functioning to evaluate, in future studies, their application in improving compliance with Non‐Invasive Ventilation (NIV) in critically ill patients. Twenty healthy volunteers underwent four 10‐min experimental auditory conditions while their electroencephalographic and polygraphic activities were recorded: Resting‐state, 6‐Hz BB, 6‐Hz monaural beats (MB), and random noise (RN). Frequency analysis and analysis of lagged‐phase connectivity were computed through eLORETA. Heart rate variability, respiratory rate, and pulse transit time were analyzed as indicators of ANS activity. 6‐Hpz BB entrained cortical activity at the beat frequency in the left cuneus and precuneus, in contrast with other experimental conditions. All auditory stimuli increased the interhemispheric lagged‐phase connectivity between auditory cortices. Contrary to MB and RN, BB induced only minimal changes in ANS parameters. 6‐Hz BB is effective in entraining cortical activity and induces only minimal changes in ANS parameters. These findings support the future use of BB as tools for increasing NIV compliance.

## 1 INTRODUCTION

Binaural Beats (BB) are a non‐acoustic perception generated within the brain when two different pure tones with the same intensity and a slight mismatch in frequency are presented separately to each ear (Oster, 1973). The amplitude of the resulting sound waxes and wanes periodically, and the frequency of these modulations constitutes the beat itself. The pulse rate of the perceived sound corresponds to the difference between the frequencies of the two original tones (Oster, 1973). The medial nucleus of the superior olivary complex is the first subcortical nucleus of the auditory pathway to receive bilateral inputs, and it seems to be involved in the BB generation (Kuwada et al., 1979). The need for a central processing for the beat perception is one of the main characteristics differentiating BB from other complex sounds, such as the Monaural Beats (MB; i.e., physical, amplitude‐modulated beats obtained through the superimposition of two sine waves at neighboring frequencies and with stable amplitude, audible individually by each ear), which are primarily processed and demodulated by the cochlea (Chaieb et al., 2015).

Since their first description in 1973 by Oster (Oster, 1973), a growing interest arose about BB and their effects on physiological and psychological processes of the human mind. Previous studies have reported the capability of low‐frequency sounds in inducing subcortical neuronal synchronization at the same frequency of the auditory stimulus, a phenomenon known as Frequency Following Response (FFR) (Marsh et al., 1970). Conflicting evidence is available about the capability of BB in generating a FFR, in entraining cortical activity at the specific frequency of the beat (i.e., auditory steady state response, ASSR), and in inducing cross‐frequency modulations.

A recent systematic review by Ingendoh et al. (Ingendoh et al., 2023) attempted to summarize the available evidence on BB‐induced ASSR, highlighting that there are highly conflicting findings on this topic. Although several studies failed to demonstrate a clear and significant effect of BB in generating an ASSR (Gao et al., 2014; Goodin et al., 2012; Lopez‐Caballero & Escera, 2017; Vernon et al., 2014), other authors have demonstrated the ability of BB to entrain the cortical activity at the specific frequency of the beat (Karino et al., 2006; Orozco Perez et al., 2020; Schwarz & Taylor, 2005).

Orozco‐Perez et al. (Orozco Perez et al., 2020) indeed demonstrated that 7‐Hz and 40‐Hz BBs used continuously were able to induce ASSR at the respective beat frequency as well as an FFR at the carrier frequency of 400 Hz. Similarly, Schwarz et al. (Schwarz & Taylor, 2005) using a carrier frequency of 400 Hz, found a BB‐induced brainwave entrainment at the 40‐Hz frequency.

On the other hand, Karino et al. (Karino et al., 2006) found that also BB in the theta frequencies were able to generate an ASSR recorded by magnetic evoked field potentials. A recent study by Hestermann et al. (Hestermann et al., 2024) found that 3‐Hz BB provoked an increase in non‐REM phase 3 sleep, demonstrating the brainwave effect of the auditory stimulus.

Finally, BB may be able to influence the autonomic nervous system (ANS) by inducing a parasympathetic activation and a sympathetic withdrawal (McConnell et al., 2014).

Due to their neurophysiological effects, several authors have investigated the role of BB as potential tools for the regulation of psychophysiological status and cognitive capacities. For instance, theta/delta band frequencies have been associated with reduced anxiety levels (Garcia‐Argibay et al., 2019), intraoperative analgesia (Lewis et al., 2004), relief from chronic pain (Gkolias et al., 2020), and improvement of sleep quality (Lee et al., 2019). On the other hand, high frequency beats, in the range of alpha, beta, and gamma, have been associated with an improvement in cognitive flexibility, memory, and attention (Garcia‐Argibay et al., 2019). However, there is conflicting evidence on this point, as summarized by a recent review by Cidral‐Filho et al. (Cidral‐Filho et al., 2024), which reported that most studies have failed to find a clear effect of BB stimulation on mood, anxiety, and cognitive performances, or have found conflicting results about it.

In this preliminary study, we aimed to assess the BB capability to promote relaxation by analyzing their effects on several neurophysiological parameters, laying the foundations for a subsequent evaluation of their utility in clinical contexts as non‐invasive tools to implement non‐invasive ventilation (NIV) compliance. To accomplish this task, we chose to employ 6‐Hz BB since, as first mentioned, low‐frequency beats seem to be those most associated with a reduction of anxiety levels (Chaieb et al., 2015; Garcia‐Argibay et al., 2019). In addition, we compared the binaural stimulation with a soundless resting condition (baseline – BS) and with two other acoustic conditions, namely 6‐Hz monaural beats (MB) and a complex acoustic stimulation composed of mixed sounds and superimposed three‐dimensional holophonic sounds (random noise – RN) in order to clarify whether the impact on neurophysiological parameters can be traced back to a generic acoustic perception itself, to its rhythmicity, or whether the central integration of acoustic stimuli represents the keystone of such a process.

The primary objective of this study was to determine whether 6‐Hz BB are able to entrain the cortical activity at the specific frequency of the beat. Secondarily, we aimed to assess the effects of BB on lagged‐phase brain connectivity within the cortical areas of the Default Mode Network (DMN) at the specific 6‐Hz frequency. Finally, we analyzed the effects of 6‐Hz BB on ANS through the analysis of multiple physiological parameters, such as respiratory rate (RR), Pulse Transit Time (PTT), and Heart Rate Variability (HRV). Based on these analyses, we would like to understand whether binaural beats can induce physiological changes in brain activity and/or ANS in order to test, in future studies, their potential application in improving compliance to NIV in ICUs.

## 2 MATERIALS AND METHODS

### 2.1 Study population

In this study, we enrolled 20 adult (≥18 years) healthy volunteers (10 men and 10 women; age range 25–53 years; mean age 31.35 years) between January 2021 and January 2022. Subjects were excluded from study participation if they met at least one of the following conditions: left‐handedness; clinical history of medical, psychiatric, or neurological diseases; major hearing loss; intake of drugs with a known effect on the central nervous system (i.e., antidepressants, antipsychotics, antiepileptics, hypnotics) in the 3 weeks prior to the study; electroencephalographic abnormalities. All the recordings were registered in the EEG lab of the Catholic University of the Sacred Heart in Rome, a sound‐attenuated, electromagnetically shielded, semi‐darkened room with a controlled temperature of 25°C. Each study participant took part in a single recording session during which he/she was subjected to four different auditory stimulations, each lasting approximately 10 min while lying on a comfortable bed with eyes closed.

Written informed consent was obtained from all the study participants. The study conformed to the Declaration of Helsinki, and the study was approved by the ethics committee of Fondazione Policlinico Universitario “A Gemelli” IRCCS–Rome.

### 2.2 Study procedures

#### 2.2.1 Auditory stimuli

Each participant underwent four different auditory experimental conditions: BS, MB, BB, and RN.

Two carrier pure sine tones were differently matched to produce monaural and binaural stimuli through Gnaural software (Gnaural version 1.0.20110606 for Linux – Copyright© 2003–2011 Bret Logan), downloadable at http://gnaural.sourceforge.net/. Resulting files, each lasting 10 min, were exported in uncompressed Waveform Audio File Format (sampled at 44100 Hz, 32 bits per sample) and each one was analyzed for quality and consistency using Audacity software (Audacity version 2.1.2 copyright© 1999–2015 Audacity Team) for Linux. Finally, spectrum analysis, phase shift, and loudness checks were performed for each file.

During all the electroencephalogram (EEG) recordings, each file was loop‐reproduced using a laptop PC with a solid‐state hard disk and closed‐back ear headphones (SHURE SRH240A: Frequency range 20 Hz–20KHz, Impedance 38 Ohm, Sensitivity 107 dB/mW, 2 m cable length) in order to guarantee at least 10 min of continuous unbiased acoustic stimulation. The laptop PC reproducing the stimuli was kept as far as the headphone cable length could afford (1.5 m minimum), far from EEG electrodes, placed on a heat‐dissipating surface, and battery‐operated in order to minimize electrical interference (i.e., electrical hums), with no fan or other moving parts operating.

Monaural stimulation was achieved by applying the same amplitude‐modulated signal to both ears simultaneously (carrier frequency 240 Hz, left–right phase shift: 0 ms) in order to obtain a beat frequency of 6 Hz. Conversely, during the BB stimulation, two carrier pure sine tones of 243 Hz and 237 Hz with equal starting phase and a slight mismatch in frequency (left–right phase shift: 2 ms) were presented separately to each ear, in order to generate a 6‐Hz beat. The pure original tone with lower frequency was always presented to the right ear. Output sound was calibrated to be presented at 70‐dB sound pressure level at each ear using a Sound Level Meter by Gain Express (http://www.gainexpress.com). The RN stimulation was generated through a file containing mixed sounds (i.e., voices and noises from an Italian street market) in order to generate an attentive response in the listeners. No iterative patterns for noise, sound, or frequency were used. Loudness was adjusted to maintain a near‐flat shape. The sequence of the experimental conditions was as follows for each study participant: BS, BB, MB, and RN. Each acoustic condition lasted between 10 and 12 min, for a total experimental procedure duration of 40–48 min, and each experimental session was played continuously, without any interruption between one auditory condition and the next.

#### 2.2.2 Polysomnographic recordings

Electroencephalographic and polygraphic recordings were registered through a BrainQuick© System Plus Evolution digital EEGraph (Micromed S.p.A., Mogliano Veneto, TV, Italy) for each study participant during each experimental condition (i.e., BS, BB, MB, and RN). The montage of the EEG was carried out according to the 10–20 system, positioning 19 scalp leads (recording sites: Fp1, Fp2, F7, F3, Fz, F4, F8, T3, C3, Cz, C4, T4, T5, P3, Pz, P4, T6, O1, O2). Reference electrodes were placed on the linked mastoids. This montage was considered adequate for the frequency and connectivity analysis since several studies have demonstrated that 19 scalp electrodes are sufficient to allow the eLORETA software to localize the spatial locations of intracranial sources, especially when resting‐state EEG rhythms are analyzed, as these rhythms are widely represented throughout the entire human cerebral cortex (Lanzone et al., 2020; Thatcher et al., 2014). Furthermore, we decided to use a standard 19‐electrode montage for source analysis as it is a simple way to record EEG traces in clinical settings, especially in Intensive Care Units (ICUs), where the BBs are ultimately intended for use.

The impedances were checked to be identical and were kept below 5KΩ before starting the recordings, and they were checked twice, before and after each participant recording. The sampling frequency was 256 Hz; A/D conversion was made at 16 bits; the pre‐amplifiers amplitude range was ±3200 μV, and low‐frequency pre‐filters were set at 0.15 Hz. The polygraphic montage included electrocardiogram (EKG), sensing of respiratory effort through a plethysmographic chest strap, and peripheral hemoglobin saturation with a pulse oximeter. The EKG electrodes were positioned according to a modified Lead II derivation (right shoulder negative, left lower torso positive).

### 2.3 EEG data analysis

The raw EEG signal was initially processed using the EEGLAB toolbox (Delorme & Makeig, 2004). Notch (50 Hz), low‐pass (70 Hz), and high‐pass (0.53 Hz) filters were applied to the EEG signal. Subsequently, electroencephalographic and polygraphic traces were decomposed using independent component analysis (ICA) (Delorme & Makeig, 2004) by means of the RUNICA script from EEGLAB. After visual inspection of individual components, electrocardiogram, pulse oximetry, and other components without visible neural activity were removed. Subsequently, the processed recordings were visually inspected for detection of remaining artifacts and blinks; muscular activations, movements, or other visible artifacts were manually removed. To delimit periods of the electroencephalographic traces compromised by artifacts, two markers were placed on the EEG raw, at the beginning and at the end of each artifact. The analysis of the EEG traces was performed per segment, choosing only segments of the EEG traces not comprised between markers. At least 280 s of EEG artifact‐free recording, not necessarily consecutive, were analyzed for each subject and for each experimental condition. The average time analyzed was 657.7 ± 79.2 s, 532.9 ± 119.0 s, 511.0 ± 120.7 s, and 533.2 ± 112.3 s, respectively, for the BS, BB, MB, and RN conditions. The resulting EEG intervals were exported into American Standard Code for Information Interchange (ASCII) files, which were then imported and analyzed by means of the eLORETA software, version 20,201,109 (Pascual‐Marqui et al., 1994).

### 2.4 Frequency analysis

The eLORETA software was used to trace the topographical sources of brain electrical activity, since this software is able to localize the cortical sources of bioelectrical activity in the brain, producing a three‐dimensional tomography in which the localization of brain signals is highly reliable in terms of spatial localization of brain areas, albeit with low spatial resolution (Pascual‐Marqui et al., 1994). For sources analysis, the eLORETA first converted the scalp activity into current source density maps (for details please refer to https://www.uzh.ch/keyinst/loreta.htm). The 6‐Hz EEG frequency, corresponding to the 6‐Hz frequency of the BB, was analyzed through the Fast Fourier Transform (FFT) algorithm provided by the eLORETA software, with a 2 s interval on the EEG signal. For each participant, the EEG activity during the BS resting condition was compared with those obtained during each acoustic experimental condition (BB, MB, and RN).

### 2.5 Connectivity analysis

Electroencephalographic functional connectivity was computed through lagged‐phase synchronization, since this measure analyzes the lagged connectivity components after the exclusion of the instantaneous connectivity components, which are often derived from non‐physiological artifacts such as volume conduction and low spatial resolution (Pascual‐Marqui et al., 2011). Due to these properties, lagged‐phase synchronization has been widely used to analyze connectivity in clinical physiology (Canuet et al., 2011).

To evaluate cortical connectivity, six Regions of Interest (ROIs) were defined, corresponding to the auditory areas included within the DMN, by employing the ROI‐maker #2 option of the eLORETA software (Table 1). The ROIs were chosen basing on the study performed by Madoux et al. (Maudoux et al., 2012), who analyzed the Magnetic Resonance Imaging (MRI) “resting‐state” connectivity patterns of the auditory network of patients with tinnitus and healthy controls. Since EEG has a lower spatial resolution than MRI, we assembled different areas considered in the study by Madoux et al. to form a single ROI (Grech et al., 2008). We chose the ‘single nearest voxel’ option, where each ROI consists of a single voxel, the closest to each seed. The eLORETA software computed the source reconstruction algorithm (Pascual‐Marqui et al., 1994) and the lagged‐phase synchronization values between all the ROIs (a total of 15 connections). The same blocks of EEG traces used for frequency analysis were used to perform connectivity analysis.

**TABLE 1:** A list of the regions of interest and the corresponding Brodmann areas considered for the connectivity analysis.

	Region of interest	Brodmann areas	Talairach coordinates		
			*x*	*y*	*z*
1	Superior and transverse temporal gyrus, Insula (right)	22, 41, 42, 44	5, 3	‐2, 7	1, 1
2	Superior and transverse temporal gyrus, Insula (left)	22, 41, 42, 44	−5, 2	−2, 7	1, 1
3	Precentral gyrus (right)	4	5, 6	−2, 2	3, 4
4	Precentral gyrus (left)	4	−5, 6	−2, 6	4, 7
5	Anterior cingulate cortex (right)	24	4, 0	−7, 5	9, 3
6	Anterior cingulate cortex (left)	24	−3, 9	−7, 8	8, 5

*Note*: All the regions of interest (ROIs) were defined by the eLORETA software (ROI‐maker #2 option) by employing the Talairach coordinates.

Abbreviation: ELORETA, exact Low Resolution Brain Electromagnetic Tomography.

In order to detect changes in lagged‐phase cortical connectivity, we compared the BS condition with each one of the other three experimental conditions (i.e., BB, MB, and RN).

### 2.6 Autonomic nervous system analysis

Heart rate variability was analyzed on the EKG traces obtained from the polygraphic records. Among time‐domain parameters, we analyzed the mean Heart Rate (HR), the time interval between two consecutive heartbeats (RR), the number of successive RR intervals which differ by more than 50 ms (NN50), and the root mean square of successive RR interval differences (RMSSD). Concerning the frequency‐domain variables, the low frequency (LF, 0.04–0.15 Hz) and the high frequency band (HF, 0.15–0.4 Hz) were computed by using the parametric Autoregressive Model analysis (*Circulation*, 1996). The power of LF and HF bands was expressed in absolute values (ms^2^), and the LF/HF ratio was calculated.

A manual rejection of artifacts was performed. All EKG periods with visible artifacts (i.e., extrasystoles, movements, muscular artifacts) were excluded from the analysis. The detection of R wave peaks and the measure of R‐R intervals (tachogram) were performed through a dedicated software (Rembrandt SleepView®, Medcare Automation B.V., Amsterdam, The Netherlands). Subsequently, the tachogram was converted into an ASCII file, and it was analyzed through a HRV Analysis Software (Biomedical Signal analysis Group, Dept. of Applied Physics, University of Kuopio, Kuopio, Finland) (Niskanen et al., 2004).

Pulse transit time is the time employed by the pulse pression waveform to travel along two different arterial sites (Smith et al., 1999). This parameter is an indirect measure of the entity of peripheral vasoconstriction and, consequently, it can reflect the sympathetic/parasympathetic balance (Smith et al., 1999). As previously described in the literature, we identified the starting point with the R peaks of the electrocardiogram, which corresponds approximately to the opening of the aortic valve, and the terminal point as the pulse pression arrival point detected by a finger pulse oximeter (Ding & Zhang, 2019). The detection of the R peaks in the EKG trace, of the peaks of the photoplethysmographic (PPG) signal, and the calculation of the time interval between the two markers (i.e., PTT) were performed by a dedicated software (Rembrandt® SleepView, Medcare Automation B.V., Amsterdam, The Netherlands). Subsequently, a visual analysis of the traces was performed, and PTTs derived from PPG waves with visible artifacts were manually excluded from the analysis.

Mean RR was obtained by the count of the number of breaths divided by the total time analyzed for each experimental condition. A breath was considered the interval between the beginning and the end of a respiratory effort against a chest strap, as detected by automatic software (Rembrandt® SleepView, Medcare Automation B.V., Amsterdam, the Netherlands).

### 2.7 Statistical analysis

Power spectra analysis and EEG connectivity in the 6‐Hz frequency band were compared between the BS condition and each one of the three auditory conditions (i.e., BB, MB, and RN). The non‐parametric mapping (SnPM) methodology supplied by the eLORETA software (Nichols & Holmes, 2002) was employed to perform the statistical analysis. This tool permits identifying differences in cortical activity among several experimental conditions by a voxel‐by‐voxel non‐paired *t*‐test of the LORETA images, based on the power of estimated electric current density, resulting in t‐statistic three‐dimensional images. In these images, a non‐parametric test (i.e., the Fisher's permutation test) identifies the cortical voxels of statistically significant differences for each comparison performed (Nichols & Holmes, 2002). Correction of significance for multiple testing was then performed by means of the non‐parametric randomization procedure available in the eLORETA program package (Nichols & Holmes, 2002). For each comparison, the eLORETA software calculates two T‐Level thresholds, which represent the values of T corresponding to a statistical significance of *p* < 0.01 and *p* < 0.05 (Friston et al., 1991).

Concerning the ANS analysis, categorical variables are expressed as number (n) and percentage (%), and continuous variables are expressed both as median and interquartile range (IQR) and as mean ± standard deviation (SD). The normality of data distribution was checked by means of the Shapiro–Wilk test. Subsequently, considering the non‐normal distribution of variables, a non‐parametric test, the Wilcoxon signed‐rank test, was employed to compare each of the three experimental conditions (i.e., BB, MB, and RN) to the BS condition. The significance level was set at *p* < 0.05. The statistical analysis was performed through the Statistical Package for Social Science (SPSS®) software version 20.

## 3 RESULTS

### 3.1 Power spectra analysis

**FIGURE 1:**
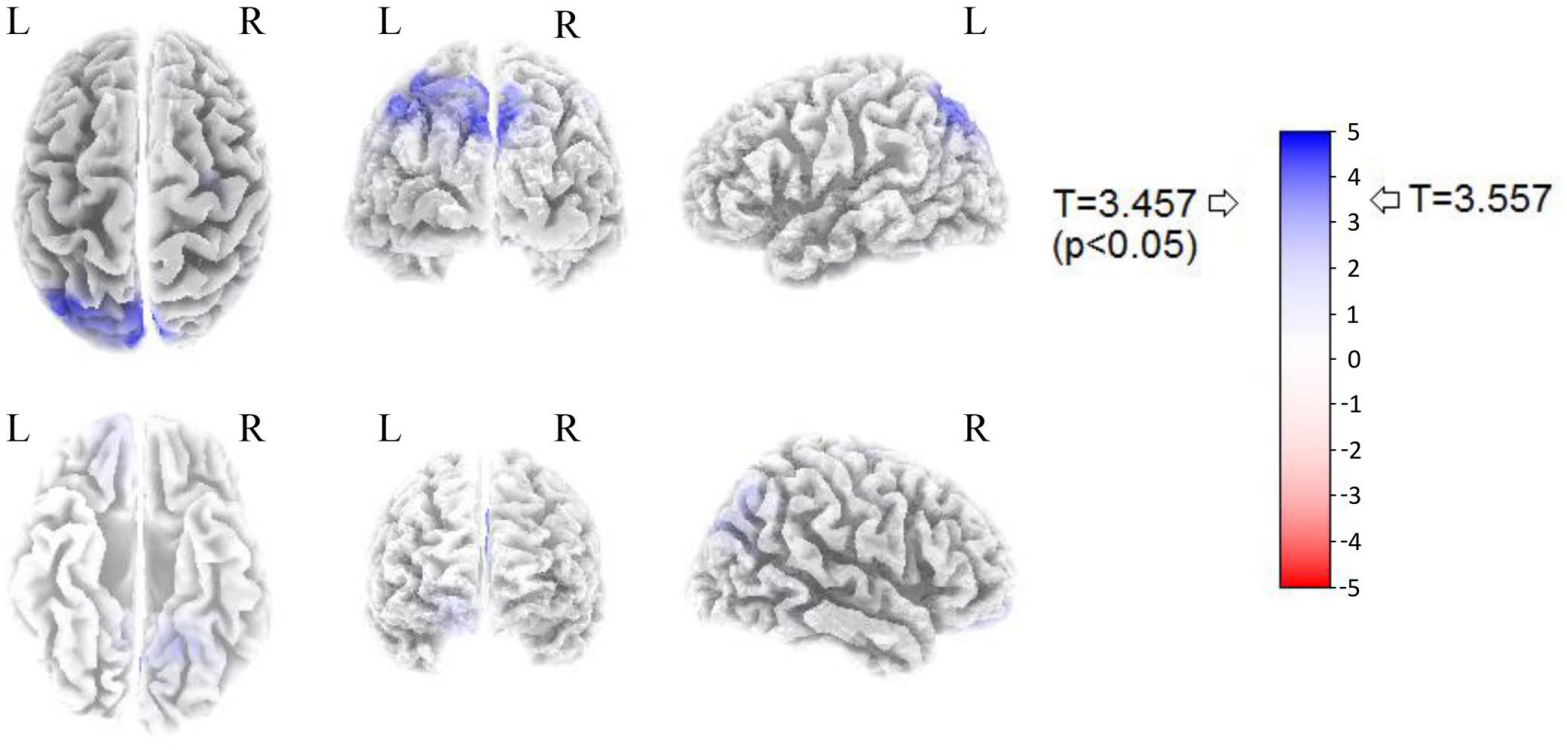
A graphic representation of the results of the eLORETA frequency analysis obtained by comparing the BS vs. the BB condition. The T value represents the threshold of significance (corresponding to a *p* < 0.05) calculated by the statistical package provided by eLORETA. BB stimuli provoked an increase in the 6‐Hz frequency in the left posterior areas of the brain, which was statistically significant in the precuneus (BA 19) and in the cuneus (BA 19). BA, Brodmann area; BB, binaural beats; BS, baseline; ELORETA, exact Low Resolution Brain Electromagnetic Tomography; L, Left; R, Right.

Thresholds for significance (*p* < 0.05) were *T* = 3.457, *T* = 3.566, and *T* = 1.807 respectively for the BS versus BB, BS versus MB, and BS versus RN analysis. Significant modifications were observed only in the BS versus BB comparison: the 6‐Hz BB stimulus induced a widespread increase of 6‐Hz activity in the left posterior parietal and occipital areas (Figure 1). The eLORETA software localized these modifications in the left precuneus (Parietal lobe: Brodmann Area, BA 19; *T* = 3.55739; BA 7; *T* = 3.53692, *T* = 3.49056, and *T* = 3.48454; *p* < 0.05) and in the left cuneus (Occipital lobe: BA 19; *T* = 3.48960, *p* < 0.05). No significant differences were observed for the other two comparisons (i.e., MB and RN vs. BS). For details, refer to Figure 1.

### 3.2 Connectivity analysis

**FIGURE 2:**
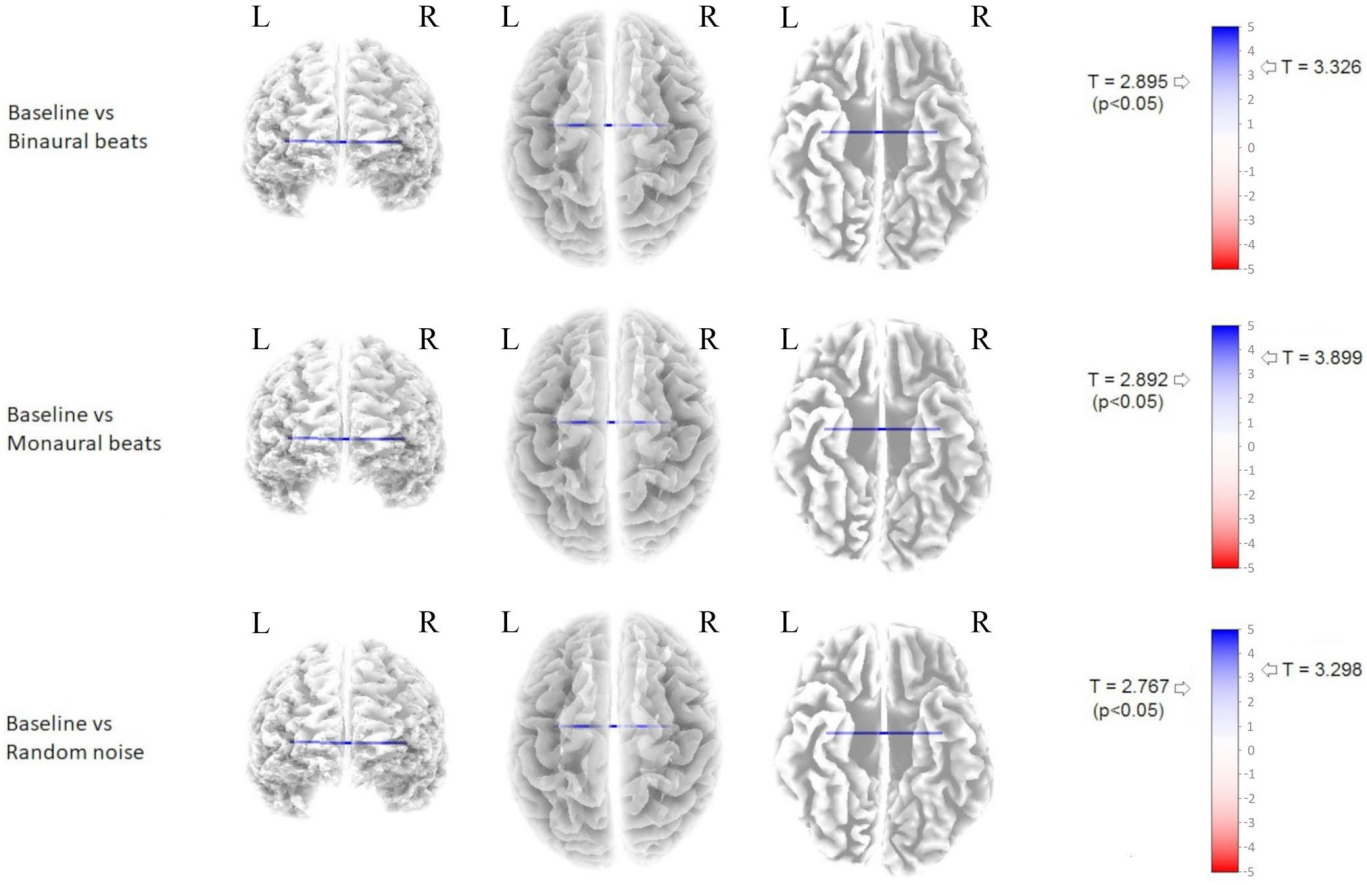
Results of the lagged‐phase connectivity comparisons between BS and each auditory stimulus considered (i.e., BB, MB, and RN) computed through the eLORETA software. All acoustic conditions were associated with a significant increase in lagged‐phase connectivity between the right and the left superior and transverse temporal gyrus and the insula (BAs: 22, 41, 42, 44). BA, Brodmann Area; BB, binaural beats; BS, baseline; eLORETA, exact Low Resolution Brain Electromagnetic Tomography; L, Left; MB, monaural beats; R, Right; RN, random noise.

Thresholds for significance (*p* < 0.05) were *T* = 2.895, *T* = 2.892, and *T* = 2.767, respectively, for the BS versus BB, BS versus MB, and BS versus RN comparisons. All the auditory conditions induced significant modifications of cortical connectivity in the selected 6‐Hz frequency. These modifications consisted of an increase in lagged‐phase synchronization between the right and the left superior and transverse temporal gyrus and the insula (Brodmann areas 22, 41, 42, 44; *T* = 3.326 for BB condition; *T* = 3.899 for MB condition; *T* = 3.298 for RN condition, *p* < 0.05). For a graphic representation of the results, refer to Figure 2.

### 3.3 Autonomic nervous system analysis

In the comparison between the BS condition and the BB exposure, we found significant differences only in one time‐domain HRV variable, the NN50, which was lower during the BB stimulation (*p* = 0.008) than during the BS. No differences in other HRV parameters, in PTT, and in RR were detected between the two conditions. Concerning the monaural stimulus, MB induced a reduction of mean NN50 (*p* = 0.002) and RMSSD (*p* = 0.048) compared to BS. On the other hand, MB was significantly associated with increased mean PTT (*p* = 0.044) compared to BS. Finally, the RN exposure was the one most associated with an alteration of HRV parameters, leading to a NN50 (*p* = 0.010), RMSSD (*p* = 0.005), and HF (*p* = 0.017) reduction and to a LF/HF (*p* = 0.028) increase compared to BS. Furthermore, the RN condition was the only one associated with changes in the RR, leading to an increase in this parameter (*p* = 0.017) compared to BS. The median and mean values of each autonomic parameter considered, and the results of the statistical comparisons, are extensively reported in Table 2.

**TABLE 2:** Results of the statistical comparisons between the autonomic parameters of each experimental condition (BB, MB, and RN) and the BS condition.

Heart rate variability	Median (IQR)	Mean ± SD	
Baseline			
Mean HR (bpm)	72.2 (66.8–81.1)	73.8 ± 10.4	
Mean RR interval (s)	0.8 (0.7–0.9)	0.8 ± 0.1	
NN50 (count)	189.0 (121.3–318.3)	219.8 ± 138.7	
RMSSD (ms)	49.3 (36.6–61.5)	49.3 ± 18.6	
HF (ms^2^)	229.7 (115.1–408.6)	300.1 ± 260.3	
LF (ms^2^)	343.1 (159.4–834.3)	491.2 ± 410.3	
LF/HF ratio	1.7 (0.9–2.9)	3.0 ± 3.4	
Pulse Transit Time (s)	0.308 (0.297–0.324)	0.311 ± 0.020	
Respiratory rate (count/min)	18.8 (16.3–20.6)	18.9 ± 3.9	

Heart rate variability	Median (IQR)	Mean ± SD	Wilcoxon signed‐rank test	
			*Z*‐test	*p*
Binaural beats				
Mean HR (bpm)	75.3 (68.5–80.8)	74.7 ± 11.3	−0.635	0.526
Mean RR interval (s)	0.8 (0.7–0.9)	0.8 ± 0.1	−0.635	0.526
NN50 (count)	137.5 (54.5–260.5)	157.2 ± 113.9	−2.670	0.008*
RMSSD (ms)	41.3 (39.2–59.0)	48.0 ± 19.4	−0.971	0.332
HF (ms^2^)	199.1 (125.9–256.0)	272.1 ± 251.1	−0.784	0.433
LF (ms^2^)	328.4 (243.6–739.1)	572.6 ± 540.5	−0.859	0.391
LF/HF ratio	1.5 (1.0–4.6)	3.2 ± 3.2	−0.448	0.654
Pulse Transit Time (s)	0.313 (0.301–0.333)	0.314 ± 0.023	−1.643	0.100
Respiratory rate (count/min)	19.0 (16.8–20.9)	18.8 ± 3.7	−0.336	0.737
Monaural beats				
Mean HR (bpm)	74.8 (64.8–81.7)	72.8 ± 12.1	−0.933	0.351
Mean RR interval (s)	0.8 (0.7–0.9)	0.9 ± 0.2	−0.784	0.433
NN50 (count)	124.5 (67.0–240.3)	151.4 ± 106.5	−3.024	0.002**
RMSSD (ms)	39.5 (32.7–57.8)	45.4 ± 17.4	−1.979	0.048*
HF (ms^2^)	180.7 (78.9–317.7)	245.7 ± 224.7	−1.755	0.079
LF (ms^2^)	377.6 (176.2–596.1)	448.9 ± 366.4	−0.149	0.881
LF/HF ratio	1.6 (1.0–4.0)	3.2 ± 3.7	−0.709	0.478
Pulse Transit Time (s)	0.316 (0.303–0.333)	0.316 ± 0.024	−2.016	0.044*
Respiratory rate (count/min)	19.7 (17.7–21.5)	19.8 ± 3.2	−1.419	0.156
Random noise				
Mean HR (bpm)	75.1 (64.7–80.8)	73.2 ± 11.3	−0.747	0.455
Mean RR interval (s)	0.8 (0.7–0.9)	0.8 ± 0.1	−0.709	0.478
NN50 (count)	150.5 (66.8–263.8)	169.8 ± 106.5	−2.576	0.010*
RMSSD (ms)	42.0 (31.0–57.6)	44.5 ± 17.8	−2.800	0.005*
HF (ms^2^)	137.5 (50.2–284.8)	201.6 ± 185.9	−2.389	0.017*
LF (ms^2^)	417.8 (215.0–877.7)	544.8 ± 429.7	−1.419	0.156
LF/HF ratio	3.6 (1.0–7.9)	6.7 ± 9.4	−2.203	0.028*
Pulse Transit Time (s)	0.313 (0.301–0.329)	0.315 ± 0.025	−1.456	0.145
Respiratory rate (count/min)	20.7 (18.5–22.3)	20.6 ± 3.7	−2.389	0.017*

*Note*: Statistical analyses have been performed through the Wilcoxon signed‐rank test. We used * to highlight significant differences with *p* < 0.05 and ** for significant differences with *p* < 0.005.

Abbreviations: HF, high frequency; HR, heart rate; IQR, interquartile range; LF, low frequency; NN50, number of successive RR intervals that differ by more than 50 ms; RMSSD, Root mean square of successive RR interval differences; RR, time interval between two consecutive heartbeats; SD, standard deviation.

## 4 DISCUSSION

The results of our study suggest that 6‐Hz BB was the only acoustic stimulus, among those considered in the analysis, capable of inducing a significant entrainment of EEG activity in the left cuneus and precuneus (BA 19). Conversely, all the auditory conditions (i.e., BB, MB, and RN) produced an increase in lagged‐phase connectivity between the auditory areas of the DMN. Finally, BB exposure was associated with only minimal alterations in autonomic parameters.

On the basis of the available literature, it is not possible to draw unambiguous conclusions on the ability of BB to entrain cortical activity. While some authors found that BB could induce cross‐frequency coupling (Gao et al., 2014), others reported that BB could entrain cortical activity to the frequency of the beat itself (Orozco Perez et al., 2020; Schwarz & Taylor, 2005), and others found no significant effects of BB on cortical activity (Lopez‐Caballero & Escera, 2017; Vernon et al., 2014). Focusing on studies that analyzed BB in the theta range, we can find previous evidence in agreement with the results that emerged in this study (Ala et al., 2018; Jirakittayakorn & Wongsawat, 2017). Ala et al. (Ala et al., 2018), analyzing the effect of a 7‐Hz BB on cortical activity, found a time‐dependent increase in cortical theta power in the bilateral temporal and parietal cortices, which was initially obtained in the left areas of the brain. On the other hand, Jirakittayakorn et al. (Jirakittayakorn & Wongsawat, 2017) reported that a 6‐Hz BB induced a dynamic, time‐dependent change in cortical theta power: After 10 min of exposure, an increase in absolute theta power was found in the fronto‐central cortex, followed by increased theta activity in the frontal and central parietal areas. However, after 25 min of stimulation, the increase in cortical theta power was found only in the right parietal lobe. On the other hand, other authors reported an effective role of theta BB in inducing cortical entrainment in prefrontal, temporal, and midline brain regions (Lee et al., 2019) while a study found no role of a 7‐Hz BB in causing alteration of cortical theta power (Goodin et al., 2012). The results of our study suggest that a 6‐Hz BB is effective in inducing an ASSR, especially in left occipital‐parietal areas, and that this effect, being statistically significant only for binaural beat exposure, is likely attributable to the central generation of the auditory stimulus. It should be mentioned, however, that several studies have shown, in contrast to the results of our study, that MB stimuli were also able to induce a brainwave entrainment to the frequency of the beat, sometimes with a greater strength than BB themselves (Becher et al., 2015; Pratt et al., 2010).

An increase in cortical theta power is often associated with a meditative state (Dobrakowski et al., 2020; Lagopoulos et al., 2009) which, on the other hand, is often associated with reduced levels of anxiety and depression (Hofmann et al., 2010) and pain relief (Carmody & Baer, 2008; Grossman et al., 2007). In particular, mindfulness meditation is a peculiar kind of meditation aiming to achieve a realistic, inclusive view of reality that requires the revisitation of the self‐concept, with reduced self‐referential processing, and the awareness of the impermanent nature of the self (Tang et al., 2015). The cortical network most involved in self‐awareness is the DMN, of which the precuneus is a key structure (Cavanna & Trimble, 2006). In order to investigate the role of the DMN in the meditation processes, several studies analyzed the DMN gray matter density (GD) of meditators compared to controls by means of Magnetic Resonance Imaging (MRI) (Berkovich‐Ohana et al., 2020; Holzel et al., 2011; Kang et al., 2013). According to the results of these studies, the GD of the parietal areas included in the DMN tended to increase after a short meditation training (Holzel et al., 2011), while the cortical density decreased in long‐term meditators, suggesting an active and dynamic role of the precuneus in meditation (Berkovich‐Ohana et al., 2020; Kang et al., 2013).

Future studies should be conducted in order to analyze whether 6‐Hz BB, by enhancing cortical theta power in the left precuneus, could induce a meditative state, similar to that achieved with mindfulness meditation, strengthening self‐awareness, reducing anxiety levels, and relieving pain. Regarding this point, a narrative review by Chaieb et al. (Chaieb et al., 2015) attempted to summarize the evidence on BB's impact on anxiety and pain perception, but evidence on BB in the theta range was sparse. Some studies reporting an effective role of BB in reducing anxiety levels were actually conducted using BB in the alpha frequency (Weiland et al., 2011), while other studies did not clearly explicate the specific beat frequency (Le Scouarnec et al., 2001; Padmanabhan et al., 2005). Furthermore, none of these studies correlated changes in anxiety perception to changes in cortical activity power.

As secondary aims of our study, always in order to test the possible role of BB as a non‐invasive and non‐pharmacological tool to increase compliance to NIV in ICU‐admitted patients, we decided to analyze the effects of BB on lagged‐phase brain connectivity within the brain cortex of the DMN at the specific 6‐Hz frequency and the impact of this auditory stimulation on different ANS parameters.

Regarding the first point, we found a non‐specific increase in lagged‐phase connectivity at a 6‐Hz frequency between auditory cortices during all the auditory conditions examined. A similar effect of BB on cortical connectivity was already reported by Solcà et al. (Solca et al., 2016), who found that alpha and theta BB increased interhemispheric coherence between Heschl's gyri in the alpha band, which was significantly less affected by respective monaural stimulation. Since in our study the increase in brain connectivity was identical for all the auditory conditions considered, we can hypothesize that the increase in lagged‐phase connectivity between the auditory cortices was not induced by the specific BB stimulation, but rather by a generic auditory stimulus, as previously reported by other authors (Reybrouck et al., 2018).

In our study, we found that BB was the auditory stimulus that presented the lowest impact on ANS, inducing only a reduction of a time‐domain, parasympathetic‐driven parameter of HRV, the NN50. In contrast, the MB and, most of all, the RN condition provoked a tangible withdrawal of the parasympathetic nervous system, evident by both HRV and respiratory parameter alterations, suggesting a concomitant sympathetic hyperactivation. On this topic, conflicting evidence is available in the present literature. Several studies reported that binaural beat stimulation induced a shift of the sympathetic/parasympathetic balance towards a vagal activation (Kelton et al., 2021; McConnell et al., 2014), while others found no changes in autonomic parameters after the administration of BB (Hautus et al., 2021; Lopez‐Caballero & Escera, 2017). The evidence emerged from this study supports the latter, reporting no clear impact of BB on ANS, and further supporting the use of BB in the intensive care unit setting as tools facilitating NIV compliance (Colombo et al., 2020). In fact, stimulus‐induced autonomic changes may alter cardiorespiratory coupling, a dynamic process based on the reciprocal interaction between respiratory and autonomic regulation of ventilation (Dick et al., 2014). Consequently, alterations of ANS parameters could alter respiratory rate (McMullan et al., 2009), leading to major respiratory distress (Nava & Ceriana, 2005) and lower NIV compliance.

This is the first study to investigate the effects of 6‐Hz BB on multiple neurophysiological parameters through a composite evaluation of EEG power, EEG lagged‐phase connectivity, and ANS analysis. The principal limitations of our study consist of the small sample size analyzed, which makes it difficult to extend the results to the general population, and the lack of a subjective assessment of the study participants after the exposure to auditory stimulations. Furthermore, we did not randomize or counterbalance the order of presentation of auditory stimuli across study participants, a strategy that could have led to biases since the effects found for BBs in brainwave entrainment may have been due simply to the transition from silence to sound (since BB were always the first auditory stimulation administered) and not specifically to the effect of BB stimulation in particular.

In conclusion, a 10‐min stimulation with 6‐Hz BB induces cortical entrainment in the left cuneus and precuneus. Lagged‐phase synchronization between the interhemispheric auditory areas included in the DMN is non‐specifically enhanced by different auditory stimuli. The autonomic nervous system is minimally affected by 6‐Hz BB stimuli. These results lay the foundations for a future evaluation of the use of BB as tools to induce relaxation in clinical settings.

## AUTHOR CONTRIBUTIONS

Conceptualization: R.M. and G.D.M.; methodology: G.D.M. and R.M.; software: G.D.M., R.M., and V.B.; validation: G.D.M., V.B., and R.M.; formal analysis: G.D.M., V.B., I.S., and J.M.; investigation: I.S., J.M., F.D.T., R.M., E.P., E.R., and R.X.; resources: G.D.M., V.B., R.M., E.P., and M.G.B.; data curation: I.S. and J.M.; writing—original draft preparation: I.S., J.M., E.P., and R.M.; writing—review and editing: I.S., J.M., G.D.M., V.B., P.A.R., R.M., M.G.B., E.P., F.D.T., G.F., and R.X.; supervision: G.D.M. and V.B.; project administration: G.D.M., R.M., V.B., M.G.B.

## FUNDING INFORMATION

This research did not receive any specific grants from funding agencies in the public, commercial, or not‐for‐profit sectors.

## CONFLICT OF INTEREST STATEMENT

None.

## ETHICS STATEMENT

This study complied with the principles of the 1964 Declaration of Helsinki and its later amendments. The research protocol was approved by the Institutional Review Board—Comitato Etico of Fondazione Policlinico Universitario ‘A Gemelli’ IRCCS—Rome.

## Data Availability

Data exposed in this paper are accessible by requiring it from the corresponding author.
